# Translation and cultural adaptation of Lithuanian version of the anterior cruciate ligament return to sport after injury (ACL-RSI) scale

**DOI:** 10.1371/journal.pone.0219593

**Published:** 2019-07-11

**Authors:** Saulė Salatkaitė, Laimonas Šiupšinskas, Rimtautas Gudas

**Affiliations:** 1 Department of Sports Medicine, Medical Academy, Lithuanian University of Health Sciences, Kaunas, Lithuania; 2 Sports Trauma and Arthroscopic Unit, Hospital of Lithuanian University of Health Sciences, Kaunas, Lithuania; Mayo Clinic Rochester, UNITED STATES

## Abstract

**Purpose:**

To translate, cross-culturally adapt and validate the scale to Lithuanian.

**Methods:**

The process of translation and cultural adaptation followed the recommendations of international guidelines. All included patients were after unilateral ACL reconstruction. Study participants completed Tegner Activity Scale, IKDC and ACL-RSI-Lt questionnaires. Reliability, construct validity and internal consistency were measured.

**Results:**

Study included sixty-five patients after ACL reconstruction: mean age 25.55 ± 6.77, mean height 180.91 ± 11.78 cm, mean weight 79.12 ± 14.88 kg and mean BMI 24.01 ± 2.90. The ACL-RSI-Lt showed excellent internal consistency (Cronbanch’s alpha 0.94). Scale scores were correlated with IKDC score (r = 0.637, p < 0.001) and IKDC subscales (r = 0.530–0.581, p < 0.001) and Tegner activity score (r = 0.303–0.493, p < 0.001). Tegner activity score before injury was significantly higher than after ACLR (6.95 ± 1.49 vs. 6.1 ± 1.37, p < 0.001).

**Conclusions:**

The ACL-RSI is successfully translated into Lithuanian (ACL-RSI-Lt). It is valid and reliable scale to evaluate the psychological impact of returning to sports in Lithuanian patients after ACLR surgery.

## Introduction

Anterior cruciate ligament (ACL) is the most common ligamentous injury sustained by professional athletes at all levels of play [1]. According to the Lithuanian Arthroscopic Surgeon Association about 950 anterior cruciate ligament injuries occur and 750 surgeries are performed every year in Lithuania. ACL reconstruction (ACLR) is the current clinical standard for patients with an ACL tear and considered to be at a high risk of knee instability [2]. An important objective of ACLR is to enable patients to return to their pre injury sport or recreational activity [3]. In order to decrease the risk of long-term deterioration of the cartilage, the reconstruction should restore normal kinematics to the knee [4].

The aim of rehabilitation after ACLR is to return an individual to his or her chosen level of activity in a timely and safe manner [5]. Recent meta-analysis has compared that 83% of elite athletes and just 60% non-elite athletes return to sport after ACL surgery [6]. Mark et al. found that 37.5% of young patients report that they feel fear to get second ACL injury [7]. Recent work has shown that fear to injury is related with low-quality movements which can be cause of second ACL injury [8]. Fear of reinjury is a powerful emotion that appears to play a role in return to sport and activity after ACLR [8]. It is becoming obvious that some athletes should require special intervention for fear of reinjury to improve function and complete a successful return to sport.

ACL Return to Sport after Injury scale (ACL-RSI) is developed to measure psychological factors associated with returning to sport following ACLR surgery. It containing 12 items subdivided into 3 domains: emotions, performance and risk assessment [9]. Developing confidence in those aspects may provide a buffer from injury-related fear and anxiety and help the athlete to resume sport participation. Also this scale was afterwards translated and validated in Dutch, Turkish, Chinese, Brazilian-Portuguese, French and Swedish languages. All versions demonstrated excellent reliability and validation [10–15]. Therefore, the purpose of this study was to translate, cross-culturally adapt and validate the scale to Lithuanian.

## Materials and methods

### Translation and cross-cultural adaptation

The ACL-RSI scale was translated into Lithuanian according to international guidelines [16]. After receiving the authors’ agreement, the scale was translated by physical therapist and translator who were all native Lithuanian speakers and fluent in English. The translations were discussed by the translators and the authors of the study, and first drafts were agreed upon. A common Lithuanian version was created using translated versions through consensus between the authors and other bilingual experts. Then the translated scale was retranslated into English by two independent native English speakers who know Lithuanian well with no medical background and have no prior awareness of the original scale. This step was to ensure that the translated version reflected the same item content as the pre-original version. In the fourth stage, the back–translated two versions of the ACL-RSI Lithuanian (ACL-RSI-Lt) scale were compared to the original version of ACL-RSI by a committee consisting of the experts. The translators and researchers then held discussions to reconcile any differences, ambiguities or language expression issues that occurred in the questionnaire and the final version of ACL-RSI was then obtained. Afterward, 10 patients (7 male and 3 female, with a mean age 23 years) with ACL injuries were asked to answer the scale and comment if they have any difficulty in answering the questions. They provided suggestions on how to rewrite the statements in order to make the language clearer.

### Participants

50 patients after ACL reconstruction surgery were involved in study. All patients were physically active a minimum of three times per week in sports that involved jumping, landing, planting, cutting, and pivoting. Inclusion criteria: participants older than 18 years, minimum 6 months after ACLR surgery, unilateral lesion of ACL. Patients who had revision surgery, multi-ligament surgery, meniscectomy or other leg injuries/surgeries were excluded. All patients received Tegner Activity Scale, IKDC and ACL-RSI scale.

### Questionnaires

The original ACL-RSI scale consist of 12 questions regarding the psychological impact of returning to sports in this population. It is centered on the three psychological responses: emotions, confidence in performance and risk appraisal [9]. Questions are initially associated with an 11 point Likert scale in the form of boxes to be ticked from 0 to 100 [17]. ACL-RSI-Lt scale items are scored from 0 to 10. The total score is obtained by adding the values of the 12 responses, then obtaining their relationship to 100 and thereafter divide from 120 to obtain a percentage. High scores corresponded to a positive psychological response.

The Subjective Knee Evaluation Form (IKDC) is used to measure symptoms, function and sports activity for people with knee disorders. The score ranges from 0 (worst possible) to 100 (best possible) and is independently produced in each subscale [18].

The Tegner Activity Scale is a numerical scale ranging from an activity level of 0 (sick leave or disability pension due to knee problems) to 10 (competitive sports on a very high level) [19]. The activity level of 10 is considered soccer, football, rugby at the elite level, level of 6 is considered the same sports but at the recreational level. Sickness and disability due to knee problems is considered as an activity level of 0 [20].

Ethical approval was obtained from the Lithuanian Bioethics Committee (no BE-2-24). Each participant signed a consent form.

### Statistical analysis

Descriptive statistics are presented as means and standard deviations. Internal consistency was measured using Cronbach’s alpha. The alpha value between 0.70 and 0.90 was considered as good, greater than 0.90 was considered as excellent [21].

In order to confirm that the scale was unidimensional, a principal component analysis was performed. Construct validity was evaluated by correlating ACL-RSI-Lt with IKDC and IKDC subscales. A Spearman correlation coefficient greater than 0.90 was considered as excellent, between 0.71 and 0.90 was considered as good, between 0.51 and 0.70 was considered as moderate, between 0.31 and 0.50 was considered as weak and less than or equal to 0.30 was considered as low [22].

Content validity was assessed by analyzing score distribution and the occurrence of ceiling and floor effects. The percentages of responders who scored the lowest or highest in each separate question on the ACL-RSI-Lt were documented. Floor and ceiling effects were considered to be relevant if more than 15% of patients achieved a score at the limits of scale [21].

All analyzes were performed using IBM SPSS Statistics 22.0 software. Statistical significance was set at p < 0.05. (http://dx.doi.org/10.17504/protocols.io.3z7gp9n)

## Results

### Translation and cross-cultural adaptation

The preliminary Lithuanian version of ACL-RSI questionnaire received no significant comments on the translation quality. The questionnaire was translated from English into Lithuanian by two native translators fluent in English. A Lithuanian version entitled “PKR-GST (Priekinis kryžminis raištis—grįžimas į sportą po traumos) klausimynas”. In order to facilitate data entry, we retained the scale of 11 boxes that were rated from 0 to 10. No changes were made after preliminary testing, as all of the patients stated that the questions were clear and easy to understand.

### Study participants

The study included 65 (39 male, 26 female) participants: mean age 25.55 ± 6.77 years, mean height 180.91 ± 11.78 cm, mean weight 79.12 ± 14.88 kg and mean BMI 24.01 ± 2.90. Minimum time after surgery among the participants was six months (43 patients), maximum– 12 months (22 patients). All participants had undergone uniliteral ACL reconstruction. Demographic information is listed in Table 1.

**Table 1:** Participants demographics.

	6 months after ACLR	12 months after ACLR	P-value
Age (yrs)	25.33 ± 6.69	26.00 ± 7.04	p = 0.781
Height (cm)	180.74 ± 11.10	181.23 ± 13.28	p = 0.713
Weight (kg)	79.58 ± 14.30	78.21 ± 16.25	p = 0.744
BMI	24.21 ± 2.73	23.63 ± 3.25	p = 0.332

### Return to sport

Activity level was measured using the Tegner Activity Scale. Also, participants were asked to evaluate their level before injury. If Tegner activity score was equal or higher than the Tegner activity score before ACL injury, it was considered that the patient returned to the pre injury level. Fourty-three of sixty-five participants (66%) returned to their pre injury level. Twenty-two patients (34%) had not reached their pre injury level. Those participants who returned to sport had higher ACL-RSI score (Table 2). Tegner activity score before ACL injury was statistically significantly higher than the Tegner activity score after ACLR (Table 3, Table 4).

**Table 2:** Differences in ACL-RSI scores between participants who returned/did not return to sport.

	ACL-RSI score Mean Score ± SD	P-value
Return to sport n = 43 (66%)	79.96 ± 12.60	p < 0.001
No return to sport n = 22 (34%)	50.19 ± 19.59	

**Table 3:** Mean values of scales and correlations between the ACL-RSI-Lt scale and other scales after 6 months after ACLR.

Scale	6 months Mean Score ± SD	Correlation with ACL-RSI-Lt	P-value
ACL-RSI-Lt	68.10 ± 21.04	-	-
IKDC	78.29 ± 10.67	r = 0.648	p < 0.001
IKDC symptom	27.54 ± 5.02	r = 0.579	p < 0.001
IKDC function	7.28 ± 1.45	r = 0.574	p < 0.001
IKDC sport	33.30 ± 4.07	r = 0.488	p < 0.001
Tegner score before injury	6.72 ± 1.49*	r = 0.368	p < 0.001
Tegner score after ACLR	6.02 ± 1.30*	r = 0.302	p < 0.001

* p = 0.002

**Table 4:** Mean values of scales and correlations between the ACL-RSI-Lt scale and other scales after 12 months after ACLR.

Scale	12 months Mean Score ± SD	Correlation with ACL-RSI-Lt	P-value
ACL-RSI-Lt	73.37 ± 20.28	-	-
IKDC	79.10 ± 14.59	r = 0.640	p < 0.001
IKDC symptom	28.05 ± 6.36	r = 0.536	p = 0.01
IKDC function	7.68 ± 1.70	r = 0.567	P = 0.006
IKDC sport	33.09 ± 5.82	r = 0.597	p = 0.003
Tegner score before injury	7.41 ± 1.44*	r = 0.438	p = 0.042
Tegner score after ACLR	6.27 ± 1.52*	r = 0.342	p = 0.02

* p = 0.007

### Reliability

The internal consistency was assessed by the Cronbach‘s alpha coefficient, wich was considered to be “excellent” if α > 0.90. Translated ACL-RSI scale based upon the strength of the correlation among the 12 items under consideration was “excellent” with a Cronbach’s alpha index of 0.94. Split-half reliability for the entire questionnaire was 0.89 and 0.92 for both halves, Guttman split-half coefficient was 0.88, correlation between first and second halves was 0.79, and Spearman-Brown prophecy coefficient was 0.89.

### Floor and ceiling effects

The overall mean score for ACL-RSI was 69.88 ± 20.78. The floor and ceiling effects of each question and the overall score were acceptable. For floor effect, the percentage of score 0 for each question ranged from 0 to 6%. The maximum permissible ceiling effect, adequate to the percentage of patients with 10 point for each question, ranged from 8 to 20%. For the overall score, 2% of participants scored below 20 and 14% of participants scored above 90. No item of the ACL-RSI-Lt was missed.

### Construct validity

Principal component analysis showed one underlying factor of the ACL-RSI-Lt with an explained variance of 62.62% and an eigenvalue of 7.51. The moderate positive correlation was observed with IKDC subjective form, subscale IKDC-symptom and weak correlation was observed with Tegner activity score before injury and after ACLR. The correlations were also moderate with subscales including IKDC-function and IKDC-sports (Table 3, Table 4).

## Discussion

The purpose of this study was to translate, cross-culturally adapt and validate the scale to Lithuanian. Lithuanian version of the ACL-RSI proved to be internal consistent, valid and reliable questionnaire for patients after ACLR. Internal consistentcy of the ACL-RSI-Lt, according to Cronbach alpha index, was 0.94 which is considered as excellent. Compared to the findings of our study, the Dutch [10] and Swedish [15] versions had the same Cronbach alpha (0.94), the Chinese [12] and French [14] versions had 0.96, the Turkish [11] version had 0.88 and the Brazilian [13] version had 0.87. All studies consolidate the strong correliation between the 12 questions of the scale, demonstraiting that 3 subscales (emotions, confidence in the performance and risk assessment) cannot be used separately. Previous studies have shown the relationship between fear and physical function performance, activity [23] and second ACL injury risk [6]. Some authors revealed that 85% of the patients reported the fear of reinjury (kinesophobia) as the main reason for not returning to sport [24]. Chmielewski and George [25] also reported that greater kinesiophobia at 4 weeks post-surgery increase the odds for not meeting advanced rehabilitation criteria at 12 weeks post-surgery. However, in literature review we haven‘t found a scale that specifically measures the psychological impact of returning to sport after incurring an ACL injury, with the exception of the ACL-RSI scale.

Some of the measurement properties of the ACL-RSI-Lt have been evaluated. We found that the mean score of the ACL-RSI-Lt was 69.43 and varied from 15–99. Also the mean scores by the gender were above 63. Ardern with authors revealed that a score of fewer than 56 points at the ACL-RSI may indicate an increased risk of not returning to the pre injury level and may help clinicians to identify at-risk athletes [26]. Either, Langford et al. [17] concluded that patients who did return to sport had a better ACL-RSI score 6 months after surgery. In our study, the scales score revealed that all patients had no increased risk to not return to the pre injury level. When comparing this study mean score with other studies, we got a little higher ACL-RSI score then Turkish (53.5), Dutch (56.1), Chinese (61.3), French (62.04) and original English versions. Likewise, in this study, no floor or ceiling effects were observed.

When compared ACL-RSI with the IKDC, the results revealed moderate correlation coefficients. In the Dutch version correlation between ACL-RSI and IKDC was 0.51 [10], in the Brazilian version was 0.58 [13], while in the Turkish version was 0.44 [11] and in the French version the correlation was 0.42 [14]. Chen et al. [12] reported a week correlation between ACL-RSI and IKDC subscales. In our study, only the correlation between ACL-RSI-Lt and subscale IKDC-sports was week. These moderate correlations can be explained by the IKDC 2000 primary developed to measure constructs, such as symptoms, limitations in function and sports due to impairment of the knee, but do not specifically evaluate psychological impact upon the return to sport. Likewise, the results of one study showed that the ACL–RSI and the IKDC are significant predictors of passing all return to sport criteria [27].

Our study has some limitations. First of all, the ACL-RSI questionnaire was not compared with other scale like KOOS scale. Secondly, the follow-up duration was too short. Future studies should examine the change in results from 6 to 12 months after ACLR. A large population of patients, following ACLR, return to the pre injury level later than 6 months.

A strength of this study is population. Included patients did not only participate in competitive sport but also took part in a recreational sports, such as jogging or cycling. Therefore, it can be suggested that the results of our study are able to represent all patients with ACL reconstruction.

## Conclusion

The ACL-RSI is successfully translated into Lithuanian (ACL-RSI-Lt). It is valid and reliable scale to evaluate the psychological impact of returning to sports in Lithuanian patients after ACLR surgery.

## Supporting information

S1 File ACL-RSI data. (XLSX)
